# Co‐creating better healthcare experiences for First Nations children and youth: The FIRST approach emerges from Two‐Eyed seeing

**DOI:** 10.1002/pne2.12024

**Published:** 2020-05-22

**Authors:** Margot Latimer, John R. Sylliboy, Julie Francis, Sharon Amey, Sharon Rudderham, G. Allen. Finley, Emily MacLeod, Kara Paul

**Affiliations:** ^1^ IWK Health Centre Halifax Nova Scotia Canada; ^2^ Dalhousie University Halifax Nova Scotia Canada; ^3^ McGill University Montreal Quebec Canada; ^4^ Eskasoni First Nation Health Centre Nova Scotia Canada

## Abstract

To achieve health, Indigenous people seek a life that balances mental, spiritual, emotional, and physical wellness, yet the scope of these four dimensions is not typically considered in the Western‐based health system. Indigenous people experience ongoing pain and hurt in all these dimensions as a result of a colonial legacy that persists in current‐day policy and care contexts. Exploring ways to support Indigenous people to embrace ways of being well and reducing chronic pain has not been a priority area in health research. This community‐based, qualitative study in four First Nations communities involved conversation sessions with 188 First Nations children, youth, parents, and Elders and 32 professionals who practice in those communities. The purpose was to gather perspectives related to pain expression, care experiences, and the strategies to improve the healthcare encounter. Thematic analysis was used to identify a more culturally thoughtful approach for clinicians to consider when First Nations people seek care. Two‐Eyed Seeing consisting of four iterative steps was used to co‐create the FIRST approach validating for community members that their perspectives were heard and providing a clinical approach for culturally safe practices with children, youth, and families. An overarching theme in the results was a clearer understanding about how pain and hurt translate into participants' health experiences and their desire to have their knowledge reflected in their health care. Participants describe experiencing pain and hurt in all four dimensions of health and from a historical, cultural, and spiritual identity, as well as from a community, family, and individual perspective. The FIRST approach captures Indigenous knowledge relating to Family, Information, Relationship, Safe‐Space, and Two‐Eyed treatment in the healthcare encounter. Considerations of this approach in clinical practice could enhance respectful and trusting relationships, knowledge exchange for better care experiences, and potentially improvement of culturally sensitive outcomes for Indigenous people.

## 1 INTRODUCTION

Indigenous knowledge of health and well‐being, amassed over thousands of years, shared from its perspectives, can significantly enhance Western‐based healthcare practices using a Two‐Eyed seeing approach. Two‐Eyed seeing invokes two sets of eyes representing the convergence of perspectives to address contemporary issues in health.^1^ Implementing this approach is anticipated to improve healthcare experiences for Indigenous people who live with pain and hurt.

Indigenous people signed treaties with settler Europeans which originally meant to recognize Indigenous rights as inherent to their way of life as First Peoples in Canada. Treaties ensured that Indigenous people access their land and its resources, like gathering medicines, hunting, and fishing, to maintain a livelihood, to live healthily, and to provide for their families and communities.^2^ The United Nations declaration of the rights of indigenous peoples (UNDRIP) extends the recognition of traditional knowledge, medicines, and cultural practices of Indigenous people, as well as their equitable and unhindered access to health care and to its governance.^3^ Indigenous peoples are in the best position to guide health providers and administrators regarding the path to wellness for Indigenous people and communities. Literature on primary healthcare disparities shows that participatory research principles and cultural tailoring of competencies must be a priority to improve health outcomes.^4^ Settler colonial practices cemented by government policies have left an intergenerational and tragic impact on Indigenous peoples’ health and well‐being.^5^, ^6^ Ongoing oppressive policies and a lack of willingness to uphold treaty and inherent rights have meant that Indigenous people have not received equitable, community‐informed, and culturally safe care.^7^ The Health Council of Canada's landmark document “Empathy Dignity and Respect: Creating Cultural Safety for Indigenous People in Urban Health Care” and other research conducted within the First Nations communities identified that Indigenous people feel fearful and disrespected and are reluctant to seek care in the healthcare system.^8^ This is especially problematic when you consider the high rates of multiple, co‐existing pain conditions experienced by Indigenous people in Canada.^9^, ^10^

Community‐led research has shown that First Nations community members, including youth, have reported that it is their perception that they wait longer than others in emergency room situations, make repeat trips for the same issues, and do not get referred for specialized health treatment.^6^, ^10^, ^11^ Higher rates of adverse health outcomes and lower rates of referral to specialist care are compounded by the fact that primary care focuses on episodic and acute care management rather than chronic disease care.^4^ Improvements to primary health care are imperative for First Nations community members where services are under‐funded and coordination between primary health and provincially funded hospital and specialty care may be poor.^4^, ^12^ The inadequate structures, practices, and jurisdictional challenges between provincial and federal policies hinder improved health outcomes for Indigenous peoples.^12^ Browne et al suggest a framework designed to reduce what she terms structural violence and in turn improve health outcomes for Indigenous populations: trauma and violence‐informed care and inequity‐responsive care.^12^

In a recent community‐based study comparing First Nations and non‐First Nations children and youth cohorts, the First Nations group had significantly higher diagnoses rates of almost all types of physical pain conditions, most notably chronic ear issues, yet First Nations children were significantly less likely to visit an ear, nose, and throat specialist.^10^ Circumstances such as these may lead to a delay in seeking care, a perception of a lack of worthiness of care, a lack of treatment, and, consequently, persistent chronic or fatal conditions that impede the optimal spiritual, mental, physical, and emotional healthy development for individuals, communities, and nations.

Our aims of this study were to gather the healthcare experiences, pain perspectives, and the strategies to improve the healthcare encounter for First Nations children and youth and health providers from four First Nations communities. This study was part of a larger study that gathered knowledge related to Indigenous youth expression of pain through art^6^ and healthcare utilization data identifying childhood pain diagnoses, occurrence, and referral rates.^10^ All three components of the study were completed in the same geographic region served by a pediatric health center in Eastern Canada.

## 2 METHODS

### 2.1 Design

The study used a Two‐Eyed Seeing approach coined by Mi'kmaw Elders Albert and Murdena Marshall, indicating that both Indigenous and Western knowledge should be considered equally beneficial to co‐create new learnings that will be relevant to support the health and well‐being of Indigenous peoples.^13^

This qualitative investigation used a community‐based, participatory action methodology embedded in the Two‐Eyed Seeing approach. This consisted of ethnographic techniques including one‐to‐one interviews and conversation sessions held at the convenience of the participants.^14^ The knowledge was protected using the communities’ protocols and the Ownership, Control, Access, and Possession (OCAP) Principles.^15^ Community member consents and raw data were maintained in a locked cabinet in participating First Nations health centers with copies kept confidential at the partnering pediatric health center. This study involved community members from three Mi'kmaq and one Wolastoq First Nations and health providers and professionals who work in the communities. The research protocol received approval from Mi'kmaq Ethics Watch, a regional Mi'kmaw ethics process coordinated through Cape Breton University (CBU) in Nova Scotia, and by the Mi'kmaq Confederacy of Prince Edward Island for PEI Mi'kmaw communities, as well as by the IWK Health Centre Research Ethics Board.

### 2.2 Setting and sample

The study was located in Mi'kmaw and Wolastoqey regions with populations ranging from 450 to 5000 community members. All communities had a community health center and were within an hour's drive of a regional hospital. A convenience sample using word of mouth and signs placed in the schools and health centers was used to recruit the youth (8‐17 years of age), Elders, parents, teachers, and professionals living and working in each community. Participants were provided with a gift card to acknowledge their participation. Elders received an honorarium for their contribution.

### 2.3 Procedure

First, a relational approach was used to establish and then maintain the community‐based participatory action methodology between the clinical research team and community Elders, Health Directors, and other representatives from each community (nurses, teachers, etc). This was done to ensure a Two‐Eyed Seeing knowledge‐gathering approach including design, implementation, interpretation, and sharing process. For example, taking the time to share food is a relational building process and snacks were provided for each session to establish trust and comfort with catering arranged from local community vendors. Another important component was hiring research assistants from each community to further the trust‐building process. One semi‐structured conversation session took place in all four communities with all members of the five community groups (Elders, parents, teachers, clinicians/professionals, and children/youth). Interviews and conversation sessions took place in the school, community center, and Elder's homes. Community members were consulted on question development, and sample questions are provided in Box 1. Data reported include participant demographics, interview, and conversation sessions (individual and group). In a previous study by this research team, it was learned that in the Mi'kmaw language, there is no translatable word for “pain,” but there is one for hurt.^11^ The word “hurt” is not a translation of pain in Mi'kmaq, but it is an interchangeable term that best embraces the concept of pain, especially for a Mi'kmaw‐speaking person. Therefore, in this study, both the terms, pain and hurt, were used in the conversation and interview questions.

BOX 1 Are there things you do to make it better, reduce the pain?

If a new health clinician was joining your Health Centre what should they know about working in your community?

### 2.4 Data collection and interpretation

The demographic data for the children and youth captured grade, sex, pain frequency, type, and cause, as well as strategies to relieve the pain. Grade is reported as it was believed to align better with Indigenous perspectives in child development in that it is a gradual process. The Mi'kmaq view of child development as an ebbing process evolves in a circular direction that transcends exact time and space, like age. Child development integrates emotional, physical, mental, and spiritual dimensions of a child manifested as learning, cultural knowledge, and well‐being simultaneously.^16^ The adult demographic sheets (completed by parent participants, clinicians, and Elders) were limited to age range, ancestry, and years of experience working/helping in the community. The sessions with children, parents, health clinicians, and professionals were primarily in English; they were audiotaped and transcribed verbatim. The interviews held in Mi'kmaq were transcribed in both the spoken language and translated to English for analysis purposes. The community members from the Wolastoq community all chose to speak in English.

Two‐Eyed Seeing was the overarching approach used with the following four iterative processes: (a) co‐learning, (b) knowledge scrutinization, (c) knowledge validation, and (d) knowledge gardening.^17^ Knowledge gardening is the active implementation of the research findings to address the research questions and needs of the community.

The first step, co‐learning, commenced with prestudy community engagements and members’ validation of the relevancy of methods and conversation session questions. The second step, the knowledge scrutinization process, involved using both a Western clinical and Indigenous lens, with investigators (ML, JRS, SR) and the community liaison who is a First Nations nurse research coordinator (JF) using open coding for the themes with consistent patterns emerging. Thematic analysis was used to probe the information generated in each of the sessions and is considered a foundational approach that draws on different epistemologies to identify, analyze, organize, and report themes.^18^

Themes were reviewed with the community research teams (Health Directors and clinical representatives) and the Elder advisors. Knowledge validation of the themes was gleaned iteratively from transcribed data and confirmed with the participants at a second community engagement session. The fourth step, knowledge gardening, is about meaningful sharing and application to address the health disparities in the communities.

## 3 MAIN FINDINGS

### 3.1 Demographics

In total, there were 220 participants in this study, 146 were children and youth, 25 were parents, 18 Elders, and 32 adult professionals (teachers [n = 14], health professionals [eg, physicians, nurses, and psychologist; n = 12], and others [eg, community police and healers; n = 6]). Demographic data were collected from all participants. Child and youth participants ranged from grades 1‐12: grades 1‐6 (n = 74), grades 7‐9 (n = 31), and grades 10‐12 (n = 38) [Three participants did not respond]. One Elder chose to withdraw for undisclosed personal reasons (17 Elders responses were analyzed).

**FIGURE 1:**
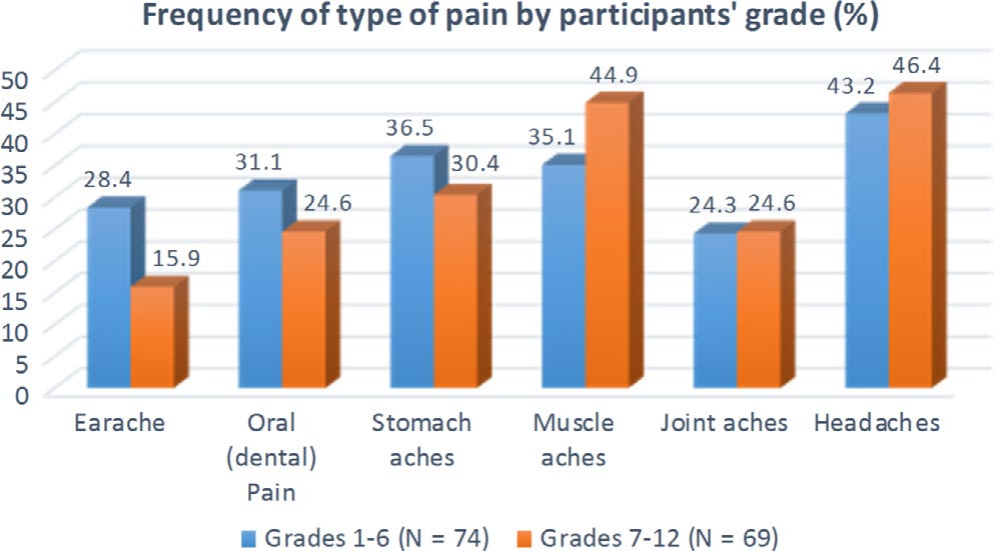
Frequency of pain experienced by child/youth participants when prompted “Yes” or “No”

By self‐report, pain frequency and type included headaches and stomach aches as the most common experience by both groups of young participants, then muscle aches common for middle and high school youth, followed by oral and ear issues common for elementary school children (Figure 1). For the child/youth participants, common causes of the pain and hurt included injuries incurred playing outside and from sports or by falls. In regard to management strategies, 55.5% (n = 81) said they take some type of medicine, and others said they lie down (68.5%, n = 100) or smudge (n = 8.2%, n = 12), while 51.4% (n = 75) indicated they distract themselves. Approximately 47.3% (n = 69) said they seek care from a nurse or physician, and another 30.8% (n = 45) hide their pain.

### 3.2 Conversation sessions

The conversation sessions for each of the five groups are themed together due to overlap across the content, yet each participant's group designation is identified when quoted. Four of the main questions included: “What does the term pain and hurt mean to Indigenous People?” “How do Indigenous People express pain and hurt?” “What do you do to manage the pain?” And “what should health clinicians know?” The results from these main questions follow here, and then, the overall themes are described.

Participants consistently responded that pain and hurt are experienced within the four dimensions of the Indigenous health perspective: mental, spiritual, physical, and emotional. Emotional hurt was the prominent experience and believed to stem from a combination of intergenerational trauma and social determinants of health. Some participants noted that there is a lack of support from the Western care system to acknowledge the spiritual and emotional care aspect. This was summed in one Elder's comment “if we only treat one thing such as our body, and our spirit isn't healthy and our minds and thoughts aren't healthy, then how can you be healthy?”

#### 3.2.1 What does the term pain or hurt mean to indigenous people?

Hurt was described from an *historical, cultural, and/or spiritual identity, and community, family,* and *individual* perspective*.* Comments categorized as *Historical* related to the Indian Residential School (IRS) experience, with Elders describing their teeth being extracted without anesthesia when they were children. One Elder stated “I was a happy person before the IRS,” and another Elder noted they were “loved and cared for by parents” before IRS. Participants identified “generations of dental pain” and “they are just used to it.” *Cultural* and *spiritual* pain was described as a “loss of language,” cutting of hair, and centralization and relocation of communities. There was a sense of “*community* hurt” and hurt between people “you got hurt or someone hurt you.” *Community* hurt was described by one Elder “you feel the pain of your sibling or your parents or other people that are very close to you.” *Family* hurt was evident when a loved one passed away, and *individual* pain and hurt occurred between people and included emotional, mental (ie, bullying), physical (ie, sports, fighting, and injuries), and *self*‐harm (ie, Cutting self, substance abuse, and suicide).

Elders expressed concern about the hurt that children today are experiencing with one Elder stating, “I can't imagine how sometimes children today are in the same situation, they carry fear…” “there may be fighting going on in the house or drinking, yeah, or abusive situations.” One youth commented that the “doctor should know that people have pain everyday.” Elders who spoke about pain perspectives from cultural and historical practices said they had witnessed a shift in how increasingly pain is self‐treated by younger generations. Pain perspectives were described as evolving within the current generations because of the colonial residue caused by IRS, the 60s scoop, and the ongoing intergenerational effects with current generations but also in how pain is treated in the Western‐based health care.

#### 3.2.2 How do indigenous people express pain and hurt?

An exceedingly common response across all groups was that children hide their pain and are stoic and quiet about their hurt and that this is a learned behavior. One Elder said, “I’m very tolerant and I don't complain too much about pain. I kind of keep it to myself, that's what I learned.” Youth stated they “don't want to look like a sook,” they “suck it up” and “sometimes they try toughening it out, sometimes they hide it.” Another youth shared, “It's hard to describe.” Being quiet was described as a learned behavior, one parent said “my dad said ‘don't let people know that you're hurt… (it's) ok to cry when you're alone.” There were several comments by all groups that “Pain leads to anger” and another youth stated they “feel sad when pain won't go away.” There was also a thread theme that holding it in is not healthy, for example, one clinician said “if you hold in your cry, it's going to make that pain build up more and more” leading to both emotional and physical pain. Participants also commented they did not want to share their hurt with friends and family because they did not want to burden them, as they were experiencing pain too, which is another example of family and community pain.

Expression was described as gender‐based, with it being more acceptable for girls, “girls more emotional, girls cry more.” Participants noted that those who identify as a boy are socialized to be “tough” with quotes including: “boys are manly,” “I feel like boys can't cry,” “boys can take the pain,” and “never heard of a big man cry before, just a little baby,” and one female youth stated, “when I was a kid dad said it is ok to cry but not the same response to my brother when he was hurt.” However, it emerged that girls as adolescents also acquire and assume the more stoic approach to pain as they get older which trumps the gender expectation of their younger years. The female youth recognized the importance of coping with pain, but they were more vocal, and they shared more about their experiences than their male counterparts.

Elders described “seeing” the pain, behaviorally in children and youth, “see it in their face,” how they walk, their eyes, avoiding eye contact, and how they isolate themselves from activities and from friends and family. Elders’ comments indicated a need to understand behaviors and noted that youth “cut so they can feel it” and the “pain reminds them they are alive.” With one Elder stating “Sometimes they become angry, abusive and destructive and they don't know why they are doing that.” Elders expressed their observations about youth from a noncondemnatory manner, and they agreed that these discussions would enhance the understanding of what youth face in their struggle with pain and hurt.

#### 3.2.3 What do you do to manage the pain?

Participants identified a range of strategies to manage their pain. Youth also described isolating themselves, laying down, rubbing the hurt body part, or applying ice or heat, “walk it off,” ignore, kiss it, band‐aid, hug, and the use of Western remedies such as acetaminophen and ibuprofen. Other ways included self‐harm like cutting, substance abuse such as alcohol with one youth stating “alcohol masks the emotional pain” and reporting taking medications like opioids, and behaviors such as bullying others, anger, acting out, and addictions. Clinicians noted that there is a sense of desperateness among youth who are not necessarily understood or heard by the health clinicians or people close to them.

All participants shared how pain was also managed with traditional medicine and strategies such as distraction through cultural activities by playing, hunting, fishing, praying, smudging, and making baskets. One Elder shared how pain during natural childbirths was managed by Elders and mothers; they would huddle to provide support by chanting, sharing stories, or even just praying in soothing voices to ensure the calmest birth setting and without the use of pain medications. There were cultural pride and valor for mothers to deliver their babies naturally. Elders described an erosion in traditional approaches to pain with the implementation of health policies, hospital practices, and modern treatments.

### 3.3 FIRST approach

Five guiding themes were “gardened” using Two‐Eyed Seeing supported by consistent knowledge from each participant group when asked about what health clinicians should know when working with Indigenous people and related to: ***F*** *amily,* ***I*** *nformation,* ***R*** *elationship,* ***S*** *afe‐Space,* and ***T*** *wo‐Eyed treatment.*

#### 3.3.1 Family

Participants described that health clinicians should know that the concept of *family* includes “the community,” as one youth said the “whole rez,” consisting of a “community of parents,” extended family members, grandparents, “aunties,” “brothers and sisters,” “friends,” and “foster care guardians.”

#### 3.3.2 Information

This theme emerged from the dual process of how *community members* shared and received information from health clinicians, and how *health clinicians* shared and received information from community members. As previously noted, community members described being “quiet,” “shy,” and “stoic” about their pain or could be “angry” and swear in their healthcare interactions. Youth shared experiences where they had difficulty describing their pain and hurt, “it's hard to describe” and didn't feel believed “they didn't help me,” and another youth described “difficulty sharing pain with ER nurse and it turned aggressive.” In terms of receiving information from clinicians, one area raised was the miscommunication or misunderstandings between English and the Mi'kmaq language. Community members are receiving the information in English, but their first language may be Mi'kmaq. One Elder described an experience with a family member seeking care “She didn't understand, no. She's never heard that before…she kept looking at me and I couldn't tell her either, you know? He (physician) said, ‘Is there anyone who can speak her language? …I found someone…and helped the doctor. That was critical. I wondered how many other children come to the hospital.” Another Elder stated, “the doctor didn't understand the seriousness …she didn't have anyone there to help explain.”

One participant said, “We are told what to do. And because we did not understand English well, we could not communicate with them and tell them what was wrong. In (nation's language) we know what's wrong and how to tell each other, but you can't convey the same meaning to someone in English what is wrong with you. The only thing the person who speaks English can do is keep guessing what is wrong with them and give them medicine that matches their guess. Then it's not even the correct medicine that is given” and another stating, “Aboriginal people want to understand more about treatment but not able to ask.” There is a perception by community members that they are “treated differently because they are seen as different people” evidenced by comments such as “when Aboriginal seek treatment, they (health providers) do not look at sickness they look at how they're dressed, personal hygiene.” Another Elder said, “that's what the white man call disparity…between the Aboriginal people with the mainstream.”

Youth made specific comments about the pain scale “everyone's scale would be different,” and others said “the scale is just really inefficient…it's really stupid” and “not really accurate scale.” One youth said in relation to having a stoic pain response and the pain scale “say you were playing a sport and hurt your wrist and the doctor asks your pain 1‐10 and you say 4 but it really feels like a 9.” The whole concept of communication is based on misunderstandings and miscommunication, which further exasperates the situation that often results in frustration for the patient during that health encounter and a reluctance to return for help.

#### 3.3.3 Relationship

This principle was described as the foundation for the success of the care encounter. Clinician participants said that providers should know that it “takes time to develop a relationship” others adding “Listen, listen, observe and listen” and “sometimes it doesn't happen right away.” Noting the child and youth needs comfortable surroundings to feel safe from the start. And another clinician said they have success when they “take a permissive approach… instead of ‘I am going to touch you say I would like to examine you, is that ok?” Successful encounters described by the youth involved situations where they felt believed, respected, and could trust the health clinicians. Clinicians recognized that each person is unique, and they want to be treated individually and respectfully. Clinicians should know about “people carrying a lot of hurt from residential schools” living with intergenerational trauma. This can result in trust issues from people seeking care which may strain or delay relationships with the clinicians; emphasizing the importance of taking time to build trust. Clinicians also recommend that clinicians situate themselves at eye level with the patient and to adjust their body language in a relaxed position showing interest. Body language must be neutral, yet demonstrate empathy, respect, and care as part of the building that relationship.

#### 3.3.4 Safe‐Space

Safe‐Space was described from both a physical and geographic space and perception about the welcome‐ness community members had when they were seeking care. Elders recognized that nurses who work in the community acquire cultural sensitivity and knowledge about the community/members which enhances healthcare encounters. One Elder described a “feeling of comfort. If you're in pain if someone is compassionate or comforting me I think that's really good,” and another Elder shared “your pain is going to be worse” if it is not a safe process. Community members described activating their coping skills before seeking care because of prior negative experiences or the perception that they will not get treated for their pain. Some people shared that they felt “discrimination in the hospital and would rather manage their kids pain at home.” They “feel safe on reserve,” and their first choice is to seek care at the community health center. Clinician participants said it was equally important to avoid stigmatizing the patient by asking racialized and culturally insensitive questions related to alcohol and drug use when not relevant for the reason they are seeking care.

#### 3.3.5 Two‐Eyed treatment

Participants identified they want to benefit from both Indigenous and Western treatment options. There are medical terminology and language miscommunication issues leaving participants with the notion that they do not know or benefit from what is available. They also describe access issues and a strong desire to have healthcare resources available in the community, that is, “if the doctor knew how to cast, wouldn't have to go to the hospital.” Community members described being helped by “healers” and just having someone to talk to. Participants described various traditional medicines such as cedar, sweetgrass, muskrat root, bark, and herbs to manage different ailments, which could include pain. Elders noted the importance of recognizing the connection with the earth and other traditional ways to manage hurt evidenced by the following quotes “ask Creator, go to the woods,” “Prayers and medicine must go together. But medicine comes from everywhere. You just don't consume it, medicine can come from a leaf, or a fruit or something else.” And yet another, “Medicine can come from a song. You can listen to someone who is singing or someone who is telling a story…words are healing.” One Elder said “When I had my heart operation I did have a lot of pain and I realized that it's going to take time, but the people that I was surrounded by, my family, and the nurses, everyone that came around were part of that healing process. And that's how I got through it.” Having clinicians know and respect that healing is relational to one's ecological and community is important. Elders expressed concern that pain management which used to be more traditional and land/play‐based, now involves “chemically based” treatments.

## 4 DISCUSSION

The findings provide a new evidence‐based, culturally relevant approach for health clinicians to consider when working with Indigenous people. Elders, youth, and health clinicians who provide care in four First Nations communities acknowledge that pain and hurt dwells within all four dimensions of the medicine wheel (spiritual, emotional, physical, and mental) and from an historical, community, family, and individual perspective. These dimensions may overlap with the hurt experience of one dimension (physical or spiritual), triggering the experience in another dimension (emotional and mental), especially when the pain goes untreated. For example, in related work by this team, a physical pain diagnosis such as a chronic ear infection in childhood was associated with a mental health diagnosis in adolescence.^10^ Further evidence by Bombay, Matheson, and Anisman that those with a parent or grandparent who attended IRS being more likely to experience psychological distress reinforces this point.^8^, ^19^ The finding that Indigenous people have a stoic and quiet response to hurt is not new in the literature.^11^, ^12^, ^20^ In addition, the management of pain and hurt that includes self‐management first, with nonpharmacological strategies such as distraction and lying down, has been reported in other research with First Nations people.^11^ These strategies may not be enough to treat undiagnosed mental health conditions, self‐harm, and substance abuse identified by participants as evidence of ongoing intergenerational trauma and colonization.^21^ Knowing and acknowledging the colonizing history of Indigenous people in Canada, such as the IRS system and the impact on current‐day health and the role of healing of the entire community, family, and/or individual, is a first step to address the ongoing inequities within the healthcare system.^22^ Implementing the TRC Calls to Action is critical to address the inequities in health through capacity building and training of clinicians in Indigenous history and health. In research conducted with five Indigenous communities in Canada, five themes emerged as relevant to understanding healthcare experiences and these include colonial legacy, the perpetuation of inequalities, structural barriers to care, and the role of the healthcare relationship in mitigating harm. Indigenous participants identified that health providers lack education about the history of Indigenous people, residential schooling, and its intergenerational and long‐term effects, which is essential in their role of supporting the healthcare journey from a spiritual, emotional, physical, and mental health perspective.^23^ This is consistent with the current study findings identifying what clinicians should know to be effective providers.

The FIRST approach (**F**amily, **I**nformation, **R**elationship, **C**ulturally **S**afe‐**S**pace, and **T**wo‐Eyed treatment) provides a guide for health clinicians to consider when building relationships and partnering for better care outcomes. Family‐centered care is one of the most common partnership models of care in children's health^24^; however, a recent scoping review notes one common limitation is that “family” is not defined. Knowing who First Nations People identify as their “family” is key to understanding who is important to the community member seeking care and who to involve in the healthcare plan. Family is kinship for Mi'kmaq people which embodies the relational aspect of care; therefore, respectful communication should emulate how a family would communicate with each other. In the current study, Elders, clinicians, parents, and children consistently said that health clinicians should know how to communicate respectfully and responsibly with patients as if they are family.

There is also a sense of a cultural understanding around expression or valor around pain, which is socially expected but may translate to stoicism to non‐Indigenous people. From an Indigenous perspective, there are exceptions about showing your pain to others and an understanding that pain is recognized as a collective lived experience. There exists an accepted cultural understanding to not cause pain or burden to others, which is linked to the principle of interconnectedness of people and their health and well‐being, as shared by Elder Murdena Marshall.^13^ The cumulative volume of historical and ongoing experiences translate into living collective consciousness by a high number of individuals. For example, the collective experience of the IRS causing intergenerational trauma is also the experience of people who did not attend the schools because they understand what pain and suffering are through a complex system of nonverbal communication in oral tradition^25^ and affecting oral memory of those experiences. Individual pain is collective pain as understood by the Elders in the study, and Bombay and colleagues identify massive collective intergenerational trauma that exists across generations.^19^ This knowledge of historical trauma is essential for care planning and support in the health context environment.

Results from this study amplify the importance of clinicians knowing how to share and receive health “***I*** *nformation”* to create an affective healthcare encounter. How the information is shared and who is doing the sharing is necessary to consider. For example, Indigenous youth may not always make eye contact when they interact with clinicians. In typical Canadian communication, eye contact is a form of communicating trust and truth between two individuals, whereas Indigenous children may avoid eye contact to show respect to older or other people. An untrained clinician may regard indirect eye contact and interpret the patient is being elusive, which can generate further distrust during the process. There is growing evidence that there is mistrust of healthcare systems across several Indigenous communities^6^, ^7^, ^8^, ^11^, ^23^ recognizing how to create trustful relationships is essential.

Knowing how information may be shared is useful for respectful communication and that a translatable word for pain does not exist in Mi'kmaw language is key to understanding the complexity of the condition. Additionally, children who speak Mi'kmaq may not express their pain as expected of non‐First Nations patients; therefore, pain assessment tools like the pain scale model may not be effective during that engagement.^11^ Mi'kmaq “is a polysynthetic language with very complicated word systems of morphology with relatively simple syntax” (Inglis, p. 393).^26^ The word “pain,” a noun and abstract, does not translate into simple syntax or the common way of speaking for L’nu (Mi'kmaq) children. Children may use words that are verb or action‐based, like “hurt” in describing their symptoms.^11^ The encounter becomes a story‐based interaction that may take more time. As a clinician, the virtue of patience will determine if trust can be established with an L’nu patient, but for the patient, it is an opportunity to be listened to, which Elders believe is part of healing. The study findings also support other research with Indigenous populations noting misunderstandings with medical knowledge/jargon.^20^ Having a language speaker with healthcare knowledge would be one way to improve the navigation of the healthcare journey.^8^, ^23^

The clinician participants in this study who routinely work with First Nations people clearly describe the importance of building the ***R*** *elationship* with the child, family, and community. From an Indigenous perspective, relationships are central to the survival of family, community, and nation; therefore, they are respectfully maintained and nurtured. Doane and Varcoe (2015) have proposed a relational approach that re‐enforces this as the foundation of the optimal caring experience.^4^ Relational approaches to care infuse cultural value into healthcare expectations when treating Indigenous people and knowledge about these perspectives is unraveling through Two‐Eyed Seeing approaches, especially for pain care.

The creation and maintenance of culturally *Safe‐Space* in health care are emerging as a consistent theme in research with Indigenous peoples in Canada. Browne et al identify this as one of the four key dimensions of equity‐oriented services to support the health and well‐being.^12^ Culturally Safe‐Spaces are created when they extend a sense of belonging in both the healthcare encounter and the physical environment and this was evident in this study with participants describing their desire to seek care in their own communities.

Safe‐Space questions build trust and relatability with the child before asking diagnostic questions, which is why asking questions about the child's interests, hobbies, and favorite music are safe and neutral engagement questions. Mindful practitioners attend in a nonjudgmental way to their own physical and mental processes during ordinary, everyday tasks. Kleinman created a patient explanatory model encouraging clinicians to use more open questions such as “I know different people have very different ways of understanding illness…please help me see how you see things?” ^27^ Creating a Safe‐Space means being aware of how to ask culturally sensitive questions not in an insensitive manner such as queries related to alcohol, tobacco, or substance use when not relevant to the health condition bringing them in for care.

Similarly, practicing from a Two‐Eyed Seeing, the study highlights the way Indigenous people share their perspectives about pain and hurt and also shares with clinicians knowledge about caring and healing practices that could amplify Western care practices. Indeed, the notion of culture as medicine and the use of story, for example, has been identified as a healing treatment important to Indigenous people,^28^ thus bringing the healthcare system closer to agreed‐upon treaties^2^ and the ideal of an inclusive, trauma‐informed, holistic system of health, care, and practice.^12^, ^13^, ^23^, ^29^

## 5 OBSERVATIONS

This paper recognizes the community voices and the sensitive nature of the issues raised related to seeking care for pain and hurt. It is not the intent of the authors to perpetuate a deficit narrative but to amplify the strengths in community and how the healthcare encounter can be improved when considering how Indigenous people want to be engaged. The idea is to provide a context as to the nature of how pain may be understood and experienced from an Indigenous perspective and an approach that considers the Indigenous peoples’ history and health context. The FIRST approach provides direction for health providers to understand and be able to foster more meaningful healthcare experiences for Indigenous people.

## CONFLICT OF INTEREST

There is no conflict of interest to declare for any of the authors.

## AUTHORS CONTRIBUTION

Latimer, Margot., Francis, Julie, and Rudderham, Sharon. co‐developed the study question, designed the study, implemented the study, performed analysis, wrote the original draft of the manuscript, and revised the manuscript. Sylliboy, John, R. implemented the study, performed analysis, wrote the original draft of the manuscript, and revised the manuscript. Amey, Sharon. performed analysis, and wrote and revised the manuscript. Finley, G. A. co‐developed the study implementation and wrote and revised the manuscript. MacLeod, Emily. co‐developed the study question, designed the study, performed analysis, and wrote the manuscript. Paul, Kara co‐developed the study question, performed analysis, and wrote and revised the manuscript.
